# Coupling SIMD and SIMT architectures to boost performance of a phylogeny-aware alignment kernel

**DOI:** 10.1186/1471-2105-13-196

**Published:** 2012-08-09

**Authors:** Nikolaos Alachiotis, Simon A Berger, Alexandros Stamatakis

**Affiliations:** 1 The Exelixis Lab, Scientific Computing Group, Heidelberg Institute for Theoretical Studies, Heidelberg, Germany

**Keywords:** Alignment kernel, Dynamic programming, PaPaRa, OpenCL, SSE, SIMD, SIMT, GPU

## Abstract

**Background:**

Aligning short DNA reads to a reference sequence alignment is a prerequisite for detecting their biological origin and analyzing them in a phylogenetic context. With the PaPaRa tool we introduced a dedicated dynamic programming algorithm for simultaneously aligning short reads to reference alignments and corresponding evolutionary reference trees. The algorithm aligns short reads to phylogenetic profiles that correspond to the branches of such a reference tree. The algorithm needs to perform an immense number of pairwise alignments. Therefore, we explore vector intrinsics and GPUs to accelerate the PaPaRa alignment kernel.

**Results:**

We optimized and parallelized PaPaRa on CPUs and GPUs. Via SSE 4.1 SIMD (Single Instruction, Multiple Data) intrinsics for x86 SIMD architectures and multi-threading, we obtained a 9-fold acceleration on a single core as well as linear speedups with respect to the number of cores. The peak CPU performance amounts to 18.1 GCUPS (Giga Cell Updates per Second) using all four physical cores on an Intel i7 2600 CPU running at 3.4 GHz. The average CPU performance (averaged over all test runs) is 12.33 GCUPS. We also used OpenCL to execute PaPaRa on a GPU SIMT (Single Instruction, Multiple Threads) architecture. A NVIDIA GeForce 560 GPU delivered peak and average performance of 22.1 and 18.4 GCUPS respectively. Finally, we combined the SIMD and SIMT implementations into a hybrid CPU-GPU system that achieved an accumulated peak performance of 33.8 GCUPS.

**Conclusions:**

This accelerated version of PaPaRa (available at http://www.exelixis-lab.org/software.html) provides a significant performance improvement that allows for analyzing larger datasets in less time. We observe that state-of-the-art SIMD and SIMT architectures deliver comparable performance for this dynamic programming kernel when the “competing programmer approach” is deployed. Finally, we show that overall performance can be substantially increased by designing a hybrid CPU-GPU system with appropriate load distribution mechanisms.

## Background

The PaPaRa tool [1] implements a new method for aligning a—typically—large number of short sequence reads against a reference multiple sequence alignment (MSA) and a corresponding phylogenetic tree. HMMALIGN [2] can also be used to accomplish this task. With certain limitations, programs for *de novo* MSA such as MUSCLE [3] and MAFFT [4] can also be deployed for this purpose. However, HMMALIGN, MUSCLE, and MAFFT align short sequence reads against a single, monolithic profile that is derived from the reference MSA without taking into account the corresponding phylogeny. PaPaRa takes the phylogeny into account by calculating multiple profiles that are obtained from the phylogeny induced by the reference MSA. The short reads are aligned against each of these phylogeny-aware profiles and the best alignment for each short read is kept. Since a large number of pairwise alignments are computed (every query sequence (QS) is aligned against every edge of the reference tree (RT)), this operation dominates the runtimes of PaPaRa. Note that all pairwise alignment operations can be carried out independently. Hence, the algorithm exhibits a large degree of parallelism.

A characteristic property of PaPaRa and HMMALIGN is that they use dynamic programming algorithms for the alignment step. Dynamic programming alignment algorithms generally exhibit a time complexity of *O*(*mn*) for aligning two sequences of length *m* and *n* against each other. This can become a limiting factor when either two long sequences or a large number of sequences are aligned. Therefore, optimization and acceleration efforts typically focus on optimizing these dynamic programming kernels. Because of its generality and importance, optimization efforts have so far mainly been undertaken for the Smith-Waterman algorithm (SWA). Because of the analogies between the SWA and PaPaRa kernels, we briefly survey SWA optimization efforts.

There exists extensive literature on vectorizing SWA with SIMD instructions on general purpose CPUs: [5] for the Intel i860, [6] for the Sun Ultra Sparc, and [7-9] for Intel x86 CPUs. The above implementations deploy fundamentally different techniques for vectorizing the algorithm: [6-8] deploy SIMD instructions to speed up the pairwise alignment of two sequences at a fine-grain level. They exploit the data parallelism in the calculations of a single dynamic programming matrix (typically denoted as intra-task parallelism). However, the inherent wavefront parallelism limits the parallel efficiency of these approaches, which motivated the introduction of increasingly sophisticated methods for alleviating these limitations. Other implementations use a fundamentally different approach. They simultaneously compute multiple pairwise sequence alignments intead of vectorizing individual pairwise alignment computations. In [5], a 64-bit special purpose register is divided into four parts for simultaneously aligning a single sequence against four other sequences. This represents a straightforward application of data parallelism, generally referred to as inter-task parallelism. In other words, the basic alignment algorithm is executed sequentially but is applied simultaneously to multiple data. The authors obtained a 6-fold speedup over the sequential implementation. Recently, Rognes introduced SWIPE [9], a highly optimized inter-task vectorization approach for modern x86 architectures that uses SSE instructions and achieves a speedup of up to 6 over the fastest intra-task SSE vectorization [8].

Furthermore, several approaches have already been assessed for accelerating the SWA on GPUs. Initial efforts used OpenGL [10]. Later implementations, after the introduction of CUDA, led to the development of SW-CUDA [11], CUDASW++ [12], and CUDASW++2.0 [13]. According to [9], the most efficient CPU and GPU implementations yield comparable performance on current state-of-the-art hardware (SWIPE on a typical quad-core CPU and CUDASW++2.0 on a NVIDIA GeForce GTX 480).

Finally, we recently introduced a FPGA implementation of an earlier version of the PaPaRa alignment algorithm [14]. This hardware architecture deploys intra-task parallelism and exploits the data (in-)dependencies in this early alignment kernel version. Although the techniques employed on the FPGA can not be directly applied to the current version of the algorithm, we obtained a speedup of two orders of magnitude.

Performance comparisons between GPU and CPU kernels are generally difficult and debatable. In order to obtain an *as fair as possible* performance assessment, we deploy what we term the “competing programmer approach”. As in previous work on using accelerators (FPGA versus x86 with AVX intrinsics [15]) for computing the phylogenetic parsimony kernel [16,17], SB explicitly worked on obtaining the best possible x86 performance and NA competed with SB to obtain the best possible GPU performance. By investing a comparable amount of optimization effort and man-hours into the x86 and GPU implementations, we hope to obtain a more realistic performance evaluation for these architectures. Provided the fast x86 and GPU implementations, we can also address the problem of how to optimally use all available computational resources on a representative modern desktop to accelerate PaPaRa. Despite the analogies to SWA, the PaPaRa dynamic programming algorithm exhibits specific challenges that are associated with the fact that our alignment model also incorporates the evolutionary signal of the phylogenetic tree. Despite the fact that the SIMT version of the algorithm was tested on NVIDIA hardware, we chose to use the industry standard OpenCL instead of CUDA. While CUDA is better adapted to the specifics of NVIDIA hardware and can potentially offer better performance, OpenCL allows for using the same code on different GPUs (e.g., AMD/ATI). Moreover, OpenCL can also be used on general-purpose multi-core systems, which is particularly convenient for testing and debugging.

## Methods

### The PaPaRa algorithm

In a phylogenetic tree, known sequences of living species (extant taxa) are located at the tips. The inner nodes of the tree represent hypothetical common ancestors of these species. Since the actual sequences of the ancestors are unknown, different methods for accommodating the uncertainty of ancestral states have been introduced in the context of scoring criteria for phylogenetic trees. In PaPaRa, ancestral states are obtained via maximum parsimony (MP), a widely used optimality criterion for phylogenetic inference. Based on a fixed, given reference MSA (denoted as RA), the key idea is to find *the* phylogenetic tree which explains the data (MSA) by the least number of mutations. The ancestral state vectors are calculated using Sankoff’s algorithm [17]. Every edge (branch) *b* of the reference tree (RT) can be represented by a parsimony state vector $A_{b}=A_{b}^{1},.\ldots \;,A_{b}^{n}$, where each $A_{b}^{i}$ represents the parsimony state of the RA at site *i* (i.e., column *i*). Each entry $A_{b}^{i}$ is encoded as a bit vector. For DNA data, each bit corresponds to one of the four DNA characters: A (Adenine), C (Cytosine), G (Guanine) and T (Thymine). This representation differs from the simpler case of pairwise sequence alignment (e.g., SWA) where each element of both input sequences represents *exactly* one character and not potential, alternative character states. In PaPaRa, an individual $A_{b}^{i}$ entry can either be an A, C, G, or T, or any combination thereof (e.g., A and T). This representation is used to encode the uncertainty of an ancestral sequence state. Thus, using these bit vectors is analogous to ambiguous character representations (e.g., M representing either G or T). The bit vectors can also be used for other input data types. The current CPU versions of PaPaRa also support protein data for instance. As mentioned before, the QS are aligned against all ancestral states derived from the edges of the RT. At each edge an additional node (a ‘virtual root’) is inserted, for which an ancestral state vector is computed. When the ancestral state vector has been calculated, the virtual root is removed again and inserted into another edge. In conjunction with this ancestral state vector, PaPaRa uses an additional signal which provides information about the gap (indel) distribution in the RT. For this purpose, we use a supplementary flag (CGAP) at each site *i*. This flag is used to appropriately adapt (calibrate) the scoring function of the dynamic programming algorithm to the indel pattern as encoded in the RT and RA. The CGAP signal is calculated along with each ancestral state vector at each edge, based on the rules described in [1]. The scoring function of the dynamic programming algorithm is provided in Equation 1. Note that the default gap and mismatch penalties are different from the default values reported in [1]. Equation 1 recursively defines the score of the dynamic programming matrix cell *S*^*i*,*j*^ in column *i* and row *j* for aligning site $A_{b}^{i}$ of the ancestral state vector against site *B*^*j *^in the QS.

$$\begin{array}{lc} & \;\;CG^{i}=\left\{\begin{array}{l} 3\;\;\text{if CGAP is set for site}\;i\\ 0\;\;\text{otherwise} \end{array}\right)\\ & (GP_{\text{OE}}^{i},GP_{E}^{i})=\left\{\begin{array}{l} (4,1)\;\;\text{if}\;CG^{i}=0\\ (0,0)\;\;\text{otherwise} \end{array}\right)\\ & \;\;M^{i,j}=\left\{\begin{array}{l} 0\;\;\text{if}\;A^{i}\;\text{and}\;B^{j}\;\text{match}\\ 3\;\;\text{otherwise} \end{array}\right)\\ & \;I^{i,j}=S^{i,j-1}+3 \end{array}$$

$$\;D^{i,j}=\text{min}\left\{\begin{array}{l} S^{i-1,j}+GP_{\text{OE}}^{i}\\ D^{i-1,j}+GP_{E}^{i} \end{array}\right)$$

(1) $$\begin{array}{ll} S^{i,j} & =\text{min}\left\{\begin{array}{l} S^{i-1,j-1}+M^{i,j}+CG^{i}\\ \;\;D^{i,j}\\ \;\;I^{i,j} \end{array}\right)\; \end{array}$$

The PaPaRa algorithm has two phases: 

• *Scoring Phase*, during which scores are calculated for each QS/ancestral state pair using Equation 1. The algorithm keeps track of the currently best (lowest) alignment score and the corresponding QS/ancestral pair for each QS.

• *Alignment Phase*, during which the actual alignments are created (via backtracking) for the best-scoring QS/ancestral state pairs.

Evidently, the scoring phase accounts for the largest part of overall runtime. The scoring phase carries out *R *∗* Q *pairwise alignments for *Q* query sequences and *R* ancestral state vectors, while in the alignment phase only the best *Q* alignments are created (i.e., one per QS). Therefore, our optimization efforts focused on the scoring phase. Note that there are no data dependencies between the *R *∗* Q* pairwise alignments. Thus, they can easily be calculated in parallel on a multi-core platform.

### OpenCL programming model

OpenCL (Open Computing Language) is an open standard for parallel programming of heterogeneous systems. An OpenCL application typically runs on a host CPU and one or more GPUs. The OpenCL architecture is similar to NVIDIA’s CUDA (Compute Unified Device Architecture), which represents an extension of C/C++ for writing scalable codes on SIMT architectures (Single Instruction, Multiple Threads).

A CUDA device consists of several *Streaming Multiprocessors* (SMs) which correspond to the OpenCL compute unit terminology. The OpenCL *work-items* and *work-groups* correspond to the CUDA *thread* and *thread block* concepts. In analogy to CUDA applications, OpenCL applications consist of a *host* program which is executed on the CPU and one or more *kernel* functions that are offloaded to the GPU. The kernel code executes work-items/threads sequentially and work-groups/thread blocks in parallel. Since threads are organized into thread blocks, thread blocks are in turn organized in *grids of thread blocks*. An entire kernel is executed by such a grid of thread blocks.

OpenCL applications can access various types of memory: global, local, shared, constant, and texture. The global memory resides in the device memory (e.g., DRAM on the GPU board) and is accessed via 32-, 64-, or 128-byte transactions. Global memory is allocated on a per-application basis and can be accessed by all work-items and work-groups. To maximize global memory throughput, it is essential to maximize memory coalescence and minimize address scatter. CUDA local memory accesses occur only for some automatic variables that the compiler places in local memory, e.g., large structures or variables that do not fit into the kernel’s register space. Since CUDA local memory resides in device memory, local memory accesses exhibit identical high latency and low bandwidth as global memory accesses. Shared memory resides on the chip and is therefore substantially faster than local and/or global memory. In OpenCL, local memory is located in shared memory and can be accessed on a per-thread basis, while shared memory can be accessed on a per-(thread)block basis. Therefore, as long as there are not bank conflicts, using shared memory allows for attaining high memory bandwidth. Finally, constant and texture memory reside in device memory and are cached.

### Inter-reference memory organization

**Figure 1:**
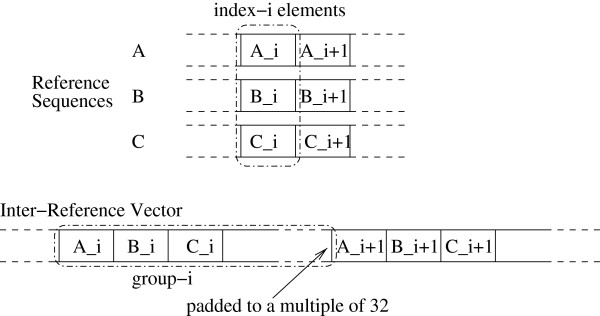
**Inter-reference memory organization.** Example of the inter-reference memory organization on SIMD and SIMT platforms. All index-*i* elements are grouped together in group *i*. Groups are padded to a multiple of 32 for performance reasons.

An inter-reference memory organization model, similar to the one described in [9], is deployed for both the SIMD as well as the SIMT implementations. The inter-reference organization facilitates the vectorization of the code on the SIMD (x86) architecture, while on the SIMT architecture it allows for coalesced global memory accesses and thereby high device throughput. In Figure 1 we illustrate this generic inter-reference memory organization approach. A certain number *W * (a work-group) of reference sequences (RS) is organized in one large array, the ‘inter-reference vector’, that consists of consecutive groups of elements. Each group contains all *R* elements for one specific position/index of the RS. The elements of a group are placed contiguously and all groups are placed sequentially in memory. In other words, the group containing the *i* + 1th elements follows the group containing the *i*th elements. The group size (number of elements per work-group) varies for the SIMD and SIMT architectures. A similar organization is used to store the dynamic programming matrix *D* (see Equation 1): each entry *D*^*i*,*j*^ consists of a group of *W * elements, while the groups in *D*^*i*,*j *^and *D*^*i* + 1,*j *^occupy consecutive memory locations. The group width (*W *) of the SIMD implementation depends on the selected integer data type and the x86 target architecture (see next section for details). For SIMT architectures, group sizes are multiples of 32 unsigned integers to ensure that the global memory is accessed in chunks of 128 bytes. Moreover, all memory transactions are aligned automatically (every address is a multiple of 128). The actual number of reference elements that a group contains is either 32 (number of unsigned integers in the group) or larger, depending on the compression of the reference entries (number of reference entries that are stored in an unsigned integer).

### SIMD vectorization

As mentioned above, the *R *∗* Q *independent alignment operations of the PaPaRa scoring phase can easily be distributed to multiple cores using threads. This corresponds to a MIMD (Multiple Instruction, Multiple Data) parallelization scheme, which scales linearly with the number of cores. Further performance improvements can be obtained by exploiting the capability of modern CPUs to simultaneously work on multiple data elements by means of dedicated SIMD (Single Instruction, Multiple Data) instructions. Based on the input data organization described in the preceding section, individual instructions of the sequential implementation (i.e., the naïve implementation of Equation 1) can be transformed into corresponding SIMD instructions. In contrast to the sequential implementation that aligns a single QS against a single ancestral reference at a time, the SIMD implementation simultaneously aligns a single QS against *W * ancestral reference sequences on each x86 core.

The number of instructions that are necessary to calculate individual entries in the dynamic programming matrix is similar for the sequential and SIMD implementations, but the memory throughput is increased by *W * since each SIMD instruction works on *W * times more data compared to its sequential counterpart. It is therefore crucial to reduce the amount of memory used by the alignment algorithm such that frequently accessed data is kept in cache. During the dynamic programming matrix computations (scoring phase), we do not keep the entire dynamic programming matrix in memory, but only a single line. This is possible because, according to Equation 1, the calculation of a matrix entry *D*^*i*,*j*^ in row *j* depends only on values of *D* that have previously been calculated either in the same row or in the preceding row *j*−1.

To make the SIMD implementation more generic, we hide the specifics of the actual SIMD instruction set (e.g., SSE) in a thin abstraction layer called a generalized vector unit, which is implemented using C++ templates. The generalized vector unit can be instantiated with different data types (16-bit/32-bit integers) and vector unit widths (8 or 4 units for 16-bit or 32-bit integers respectively when using SSE). The top-level algorithm uses abstract vector data types (integer vector of 8x16 bits) and vector operations (e.g., load data from a memory location into a 8x16-bit integer vector, add two 8x16-bit integer vectors, etc.). Here, the group width *W * corresponds to the width of the vector unit (i.e., either 4 or 8 units for 16-bit or 32-bit integer vectors) on our primary target platform, an Intel x86 with SSE instructions. This ensures that the element groups in the RS and the dynamic programming matrix *D* can be directly loaded into CPU vector registers. We implemented the generalized vector unit for Intel x86 CPUs using SSE version 4.1 intrinsics. Note that SSE version 4.1 is only required for using 32-bit integers, while for 16-bit integers SSE version 2 or higher is sufficient. We have verified that the concept of the generalized vector unit also works correctly for other vector instruction sets (e.g., ARM NEON or Intel AVX instructions; data not shown).

### SIMT inter-task parallelization

**Figure 2:**
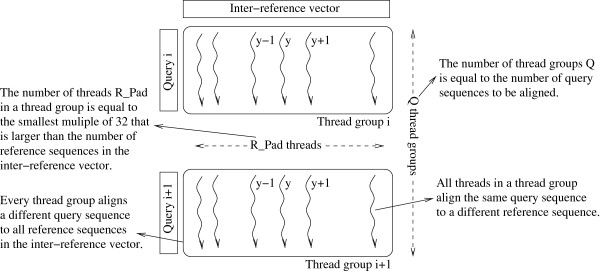
**Inter-task parallelization strategy.** Example of the inter-task parallelization strategy. Thread *y* of group *i* aligns QS *i* to RS *y*. The number of threads in a thread group depends on the number of RS in the inter-reference vector. The number of thread groups depends on the number of QS. Every thread group aligns a different QS to all RS while all threads in a thread group align the same QS to all RS.

On the SIMT platform, each alignment kernel invocation calculates one dynamic programming matrix. To efficiently execute the kernel on a GPU, every individual dynamic programming matrix calculation is assigned to a distinct thread (inter-task parallelization). An alternative parallelization scheme using intra-task parallelization would assign each task (dynamic programming matrix calculation) to a thread block, and all threads within that block would then cooperate to accomplish the task. Liu *et al.*[12] investigated both the inter-task as well as the intra-task parallelization approaches for porting the SWA to SIMT platforms. They found that inter-task outperforms intra-task parallelization. However, the intra-task approach requires significantly less device memory per thread. The intra-task approach is also appealing in cases where a large number of independent pairwise alignments need to be performed. However, in PaPaRa, each QS is aligned against a large number of RS. This alignment workload can be grouped into many 1-to-W alignments, which naturally fits the inter-task paralellization scheme. Previous experiments with the SWA by other authors showed that, if the problem at hand is such that the inter-task approach can be deployed, it consistently outperforms the intra-task approach on SIMD [9]*and* SIMT [12] architectures. The inter-task approach is more communication-efficient since frequent synchronization between threads operating on a single, shared dynamic programming matrix is not required. Threads only need to be synchronized once upon kernel termination. Therefore, we did not further investigate the intra-task approach for PaPaRa. Figure 2 depicts the inter-task parallelization approach we use here.

### Block-based matrix calculation

**Figure 3:**
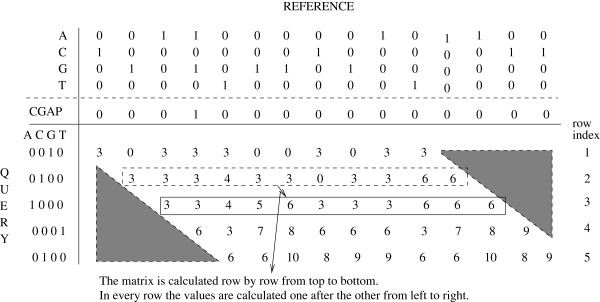
**Straightforward calculation of the PaPaRa scoring matrix.** The straightforward approach for calculating the scoring matrix consists in calculating one row after the other. In every row the values are calculated from left to right. Therefore, an entire row is calculated before proceeding to the next one.

**Figure 4:**
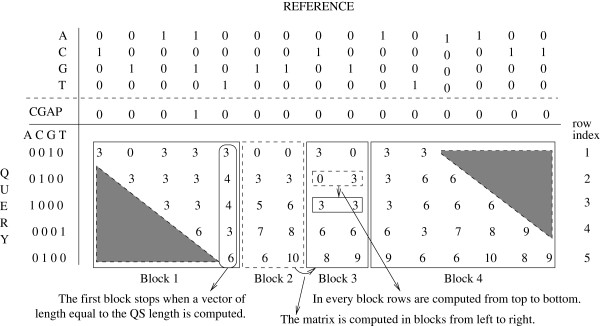
**Block-based calculation of the PaPaRa scoring matrix.** The dynamic programming matrix is calculated in blocks, starting from the left-most square block and proceeding toward the right-most square block. In every block the rows are calculated one after the other. Block 1 stops when the last row of the matrix is reached. It operates on global memory due to its size. The main part of the reference is processed in rectangular blocks (Blocks 2 and 3). These blocks are of significantly smaller size and operate on shared memory. Block 4 processes the last part of the reference sequence in global memory. Blocks 1 and 4, which operate on global memory, represent an engineering trade-off for avoiding *if-else* conditional statements in the GPU kernel that would slow down Blocks 2 and 3. To process real-world references, many more than 2 iterations over the rectangular blocks are required.

A straightforward approach for calculating the PaPaRa dynamic programming matrix is to compute one row after the other in a diagonal direction as shown in Figure 3. The memory requirements of this approach depend on the size of the inter-reference vector, since only the last matrix row needs to be stored in memory for calculating the next row. However, this approach requires off-chip global memory and does not allow for using on-chip shared memory. The main reason for this is that the size of the inter-reference vector in real-world scenarios will typically exceed the amount of shared memory available on a representative SIMT platform. Furthermore, the entire row needs to be calculated before proceeding to the next row. To alleviate these shortcomings, and to be able to use shared memory, we devised a block-based approach. The matrix cells are calculated in square or rectangular blocks of adjustable size. The block size is a function of the length of the query sequences, the amount of available shared memory, and the number of RS. Figure 4 depicts this blocked matrix calculation model.

Due to the diagonal direction of the PaPaRa matrix calculations and the limited amount of on-chip memory, it is not possible to use shared memory efficiently along the entire inter-reference vector. This means that the very first and very last parts of it need to be calculated using global memory for storing the intermediate values of the row fraction they are operating on. The part of the inter-reference vector residing in the rectangular blocks can, however, be calculated using shared memory. Using rectangular blocks at either end of the RS requires the evaluation of additional conditional (*if-else*) statements in the kernel code. Note that evaluating conditionals can substantially deteriorate GPU performance. Thus, we use global memory at either end as a trade-off to circumvent this problem and to avoid evaluating conditionals that would slow down processing of the main part of the inter-reference vector. Our approach only requires a fixed amount of global memory, irrespective of the length of the RS (stored in the inter-reference vector), since the full length RS is processed by iterating over blocks of fixed size. In the example provided in Figure 4, Blocks 1 and 4 use global memory while Blocks 2 and 3 operate on shared memory.

### Loop unrolling

An advantage of the blocked approach over the straightforward approach is that the amount of global memory required for the computation of a single dynamic programming matrix does not depend on the length of the RS. Nevertheless, code complexity increases since three nested for-loops are required to compute the entire matrix: one loop iterates over the rectangular blocks, the second loop iterates over the query sequence, and the innermost loop iterates over the reference fraction in the current block. We observed that the anticipated performance gain by using shared memory was reduced by the increased code complexity, which hindered the GPU threads from efficiently executing the blocked version of the OpenCL kernel. To solve this problem, we unrolled the innermost for-loop that iterates over the fraction of the reference corresponding to the rectangular block.

Loop unrolling significantly improved kernel performance but comes at a cost: the number of RS that the kernel can align is hard-coded. However, this limitation is not problematic, since in typical real-world scenarios users will align thousands of reference and query sequences. In this case, the amount of dynamic programming matrices to be computed is organized in groups that contain a fixed number of RS. Thus, the OpenCL kernel can be launched several times on those groups. We opted for using a fixed number of RS based upon an in-depth investigation of the impact of the shared memory size setting on OpenCL kernel performance. In our implementation, we use 15 KB of shared memory because a significant slow-down was observed when using larger amounts of shared memory. The number of RS per kernel launch is fixed (hard-coded) to 320, which allows for unrolling the innermost loop 12 times. Thus, we were able to completely remove this loop because the selected shared memory size only allows for processing 12 columns of the dynamic programming matrix in each block.

### Data compression

**Figure 5:**
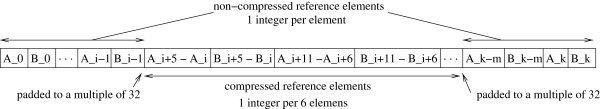
**Compressed inter-reference memory organization.** The non-compressed fractions of the vector are processed by the square blocks while the compressed part is processed by the rectangular blocks.

The global memory access pattern is also performance-critical on SIMT platforms. Therefore, we compressed the part of the inter-reference vector that is processed by the blocks in shared memory to reduce the frequency of global memory accesses. Every element (inter-reference vector entry) in the inter-reference vector requires 5 bits (4 bits for the parsimony state plus 1 bit for the phylogenetic gap signal CGAP) which allows the use of one unsigned 32-bit integer value to store 6 elements. This boils down to only two global memory accesses for every 12 elements (or two global accesses per rectangular block). Since square blocks are not unrolled, the inter-reference vector entries in such blocks are also not compressed for the sake of code simplicity, and to allow for the efficient execution of threads. Figure 5 outlines how the inter-reference vector is organized on the SIMT platform. The uncompressed initial and final parts of the vector require one integer per element while the compressed part requires one integer for every 6 elements. Note that all three parts of the vector (uncompressed initial and final parts and the compressed intermediate part) are padded to a multiple of 32 unsigned integers.

### OpenCL application

The complete OpenCL application consists of tasks performed by the CPU (host program) and operations that are offloaded to the GPU (kernel functions). The host program handles the inter-reference memory organization and compresses the input RS. Once the references have been rearranged (organized into the inter-reference vector) and compressed, they are stored in pinned (non-pageable) contiguous memory space. The query sequences are also stored in contiguous blocks, each the length of the longest query sequence. Allocating pinned memory for the query and inter-reference vectors allows for fast GPU data transfers via PCI Express. After the data (query and reference sequences) have been transferred to the global GPU memory, the host program launches a one-dimensional kernel of size *Q*∗*R*_*Padded*, where *Q* is the number of query sequences and *R*_*Padded *the smallest multiple of 32 that is greater than the number *R* of RS. Those *Q*∗*R*_*Padded* threads are organized in *Q* local groups of size *R*_*Padded*. This local, group-based thread organization simplifies the indexing of the query sequences in the kernel function. Note that the number Q of query sequences that is aligned per kernel call usually only represents a fraction of the total amount of query sequences in the input dataset, due to memory limitations.

Finally, the kernel function implements the actual PaPaRa alignment kernel. The local group index in the kernel references the query sequence while the thread index references the RS. Thus all threads in a local group align the same query sequence to all RS in device memory. The query and reference sequences are retrieved from global memory and the intermediate values (the values of the dynamic programming matrices) are either stored in global memory (first and last square blocks) or in shared memory (rectangular blocks). As described before, all global memory accesses are correctly aligned to minimize the performance impact of high-latency global memory accesses.

## Results

### GPU Performance

To assess performance of the OpenCL SIMT implementation, we used a heterogeneous system equipped with an Intel i7 2600 CPU running at 3.4 GHz (SIMD platform) and a NVIDIA GeForce 560 GPU with 336 CUDA cores and 1 GB DDR5 device memory (SIMT platform). We measured total execution times as well as GCUPS (giga cell updates per second) for kernel executions on real-world datasets.

Initially, we focus on the performance of the OpenCL kernel for different input dataset sizes. To this end, we investigate the behavior of single kernel launches with different RS lengths between 500 and 500,000 nucleotides. Every kernel launch aligns 1250 query sequences with an average length of 100 nucleotides to 320 RS. As shown in Table 1, the performance of the OpenCL implementation improves with the RS length until the peak performance of 22 GCUPS is reached. For very short RS (e.g., 500 nucleotides), kernel performance drops below 50% of peak performance. As already stated, the block-based implementation uses global memory for the first and last square blocks of the matrix and shared memory for the intermediate rectangular blocks. When the RS is short, global memory will predominantly be used for dynamic programming matrix calculations, since the square blocks almost cover the entire matrix. In other words, because of the short reference length, the potential performance gains by using shared memory are negligible, since the majority of operations is conducted on global memory. For longer sequences however, the majority of operations is carried out on shared memory and therefore improved GPU performance is attained.

**Table 1:** OpenCL kernel performance for RS with different lengths

	**Execution times**			**GCUPS**			**Speedup GPU vs**	
**Reference length**	**Seq**	**SSE(4)**	**GPU**	**Seq**	**SSE(4)**	**GPU**	**Seq**	**SSE(4)**
500	40.79	1.11	1.89	0.49	18.01	8.4	21.58	0.59
1000	90.45	2.58	2.5	0.44	15.52	14.4	36.18	1.03
5000	494.65	14.30	9.23	0.40	14.30	21.2	53.59	1.55
10000	1006.1	29.27	18.31	0.40	13.66	21.6	54.95	1.60
50000	5103.4	319.92	90.95	0.39	6.25	21.9	56.11	3.52
100000	10369	1785.8	181.31	0.38	2.24	22.0	57.19	9.85
500000	51448	9005.3	906.21	0.39	2.22	22.1	56.77	9.94

Execution times (in seconds) to align 1250 QS to 320 RS.

SIMD performance behaves differently with increasing RS length. The cumulative performance on 4 cores is highest for the short references with 500 base pairs (18.01 GCUPS) and decreases linearly to reference lengths of 10,000 base pairs (13.66 GCUPS). We observed a further substantial performance deterioration on very long reference sequences (e.g., 6.25 and 2.22 GCUPS for lengths of 50,000 and 500,000 respectively). This is due to the increased number of cache misses when longer references are analyzed. Our SIMD implementation keeps one line of the dynamic programming matrix in memory. Each matrix entry corresponds to a vector of 8x16-bit values (16 bytes). A reference length of 10,000 requires a matrix line size of roughly 160 KB, which fits into the L2 cache (256 KB per core) of the Intel i7 2600 CPU. For a reference length of 50,000, the matrix row occupies 800 KB and does not fit into the L2 cache. Therefore, most matrix cell calculations need to access the slower L3 cache (8 MB shared by all cores). The slowdown is even larger for the longest sequences under examination, where the data do not fit into L3 cache either. Currently, we do not expect these slowdowns to be problematic for real-world scenarios because current sequencing devices rarely produce reads exceeding a length of 1000 base pairs [18]. However, when future sequencing technologies (e.g., PacBio http://www.pacificbiosciences.com) are capable of generating a large number of reads longer than 1000 base pairs, then a blocking technique similar to the one devised for the SIMT implementation could also be applied to the SIMD implementation.

We also investigated the performance of the OpenCL kernel for different numbers of query sequences. We chose a fixed RS length of 5000 since this represents an average case scenario, and this is close to our GPU’s peak performance levels. Keeping the reference length constant, we launched the kernel multiple times using different query sequence numbers. The results of this performance assessment are provided in Table 2. While kernel performance is practically independent of the number of query sequences, there is a slight performance improvement up to 500 QS for the SIMD implementation. This is caused by the initial overhead required for transforming the RS into their inter-reference representation, which has to be done once for each block of *W * RS but is independent of the QS number. For more than 500 QS this initial overhead becomes negligible. As expected, the performance of the sequential implementation is completely independent of the QS number. We did not conduct any tests to examine the performance of the kernel for different average query sequence lengths, as the PaPaRa algorithm has been designed for aligning short read QS (e.g., Illumina or 454 reads) to a RA.

**Table 2:** OpenCL kernel performance for different number of query sequences

	**Execution times**			**GCUPS**			**Speedup GPU vs**	
**Number of queries**	**Seq**	**SSE(4)**	**GPU**	**Seq**	**SSE(4)**	**GPU**	**Seq**	**SSE(4)**
100	39.60	1.14	0.78	0.40	14.10	20.2	50.8	1.46
250	98.31	2.78	1.87	0.41	14.27	20.9	52.6	1.48
500	197.57	5.57	3.7	0.40	14.33	21.1	53.4	1.51
750	296.23	8.40	5.5	0.40	14.24	21.1	53.9	1.53
1000	395.59	11.20	7.4	0.40	14.28	21.2	53.4	1.51
1250	494.65	14.30	9.23	0.40	14.29	21.2	53.6	1.52

Execution times (in seconds) to align the QS to 320 RS with length 5000.

### Hybrid CPU-GPU approach

To achieve the maximum possible performance for a typical CPU-GPU system, we also designed a hybrid PaPaRa version that simultaneously uses all available cores as well as a GPU. In addition to multi-threading (available in the original implementation [1]), the CPU part of the hybrid system uses the SIMD implementation described earlier with a vector/group-width *W * = 8 and 16-bit scores. Initially, the algorithm generates and groups the RS into ‘blocks’ of *W * sequences. These blocks are stored in a work queue, and are sequentially retrieved by multiple worker threads simultaneously. Each worker thread aligns all QS against the *W * reference sequences in the block. Once all queued blocks have been calculated, the master thread resumes control and, for each QS, only re-computes the alignment for the respective best-scoring QS/RS pair to perform the backtracking step.

The GPU part of the hybrid system extends this mechanism by an additional GPU thread. In analogy to the CPU threads, the GPU thread consumes blocks from the same work queue. A key difference is that, while each CPU thread only removes one block from the queue at a time (i.e., *W * matches the native vector width of the CPU), the GPU works on a higher number of RS at a time (e.g., 320). The GPU thread can remove up to 40 blocks from the work queue at a time. The GPU thread continues to obtain and work on multiple blocks until the block queue is empty. Thereby, the CPU cores and the GPU compete for RS. For a sufficiently large number of RS this yields good load balance between the CPU cores and the GPU. This approach can also be extended for using more than one GPU.

The GPU DRAM size limits the number and the length of QS that can be transferred to the GPU at each invocation of the alignment kernel. While the number of QS per invocation has to be small enough to fit into the available DRAM when the QS are long, for short QS the number has to be high enough to achieve good performance (aligning a small number of short QS greatly reduces the performance of the GPU aligner, data not shown). The hybrid CPU-GPU algorithm dynamically optimizes the number of QS based on the available amount of DRAM and the actual QS lengths. Thereby, we ensure that each kernel invocation operates on the largest possible QS number. This dynamic load balancing allows for stable performance over different dataset shapes with different sequence length distributions (see next section).

Currently, the OpenCL implementation faces a technical difficulty on NVIDIA GPUs. Once a kernel is invoked, an internal thread of the GPU driver executes a busy-wait, apparently (the driver is closed-source) waiting for the GPU to finish. If a clFinish call is deployed on the CPU side to wait for completion of the GPU kernel, this will, in addition to the internal driver thread, cause the calling thread to execute a busy-wait, effectively wasting the computational power of two CPU cores. While CUDA offers a method for selecting between a busy-wait (for fine-grain, fast kernels) and a lazy-wait, this option does not yet exist in OpenCL. From the values shown in Table 2, we can roughly estimate that the speedup of the GPU over two CPU cores is approximately three-fold (assuming that 14 GCUPS are obtained on 4 cores) in the best case. This effectively means that wasting two CPU cores for interacting with the GPU has a negative impact on overall system performance. An analysis of the NVIDIA OpenCL library showed that the internal busy-wait implementation executes sched_yield function calls which should yield CPU cycle time to other threads. In practice, this call has no positive effect and overall system performance decreases in proportion to the number of threads that are executing a busy-wait. This busy-wait issue is a well-known Linux problem (see e.g., http://www.mail-archive.com/linux-kernel@vger.kernel.org/msg91605.html). To temporarily circumvent this issue, we use a customized shared library to replace (using LD_PRELOAD) all sched_yield calls by usleep calls. This actually yields CPU cycles to other threads at the cost of increased kernel call latency, but only of the order of a few milliseconds. This latency increase is not critical here because each GPU kernel invocation requires a few seconds to complete when the number of QS is sufficiently large (see previous section). Using this work-around, we can leverage the entire computational power of the GPU and *all* CPU cores.

### System performance

We assessed overall performance of the hybrid CPU-GPU algorithm using two representative real-world datasets. The experiments were performed on the same system as described above (i7-2600 CPU, GeForce GTX 560), using all 4 CPU cores. The first test dataset (1604.PRANK) has already been used to evaluate the original implementation of PaPaRa in [1]. This dataset contains 802 RS of length 3060 and 16,040 QS with a mean length of 100 base pairs. We did not use other datasets from the original study because the hybrid CPU-GPU algorithm targets larger datasets. The second dataset (16S.B.ALL) is from a recent study comparing PaPaRa to a newly developed algorithm [19]. The unoptimized, proof-of-concept implementation of PaPaRa performs better than competing alignment approaches on the latter real-world dataset at the cost of substantially higher runtimes. This dataset consists of 13,822 RS of length 6857 and 13,820 QS of lengths that vary between 29 and 483.

Table 3 shows the performance of the hybrid CPU-GPU algorithm on the two datasets. Column *T*_*scoring*_ provides the runtime for the scoring phase. Column *T*_*all *_shows the overall runtime for the whole algorithm, including the pre-processing of input files and the generation of the actual alignments. These pre- and post-processing steps are unoptimized sequential tasks that are performed on the CPU. The overall CPU performance is shown in column *GCUPS*_*CPU*_, which provides the accumulated performance on 4 CPU cores. Overall GPU performance is provided in column *GCUPS*_*GPU*_. On both datasets, the relative contribution of the CPU cores and of the GPU are very similar; the CPU and GPU contribute 40% and 60% of the overall GCUPS to the accumulated CPU-GPU system performance, which is shown in the last column (*GCUPS*_*all*_). All GCUPS values refer to sustained GCUPS since they include the overhead induced by load imbalance between the CPU and the GPU. Load imbalance is observed when either one of the CPU threads or the GPU finish last and require the other computational resources to wait. The sustained real-world performance of the hybrid system corresponds, almost exactly, to the performance we obtained for the synthetic benchmarks.

**Table 3:** System performance of the hybrid CPU-GPU algorithm

**Dataset**	***T***_***scoring***_**(s)**	***T***_***all***_**(s)**	***GCUPS***_***CPU***_	***GCUPS***_***GPU***_	***GCUPS***_***all***_
1604.PRANK	227.21	273.12	13.38	20.04	33.42
16S.B.ALL	12943.9	13111.4	13.87	20.00	33.87

Runtimes (in seconds) for the scoring phase and the whole algorithm.

## Discussion

Intuitively, the PaPaRa algorithms exhibits a similar complexity to the SWA, since they both are dynamic programming kernels. However, specific features for incorporating phylogenetic signal in the PaPaRa algorithm make it more challenging to implement than the SWA. In PaPaRa, the reference input sequences are parsimony state vectors. Hence, 4 bits are required for representing parsimony vector entries for DNA data. In SWA, reference input sequences are simply plain characters and not parsimony states. Thus, SWA requires only 2 bits for each nucleotide, which means that PaPaRa exhibits higher memory requirements than SWA. Furthermore, PaPaRa calibrates gap penalties by using an additional CGAP flag. This model increases the gap calculation cost and does not allow for optimizing gap-open penalty calculations as used in SWIPE [9]. The fact that the SWA algorithm can therefore be mapped in a more compact way to x86 and SIMT platforms explains the higher GCUPS performance obtained [9,13].

The PaPaRa kernel was implemented in OpenCL. Several informed design decisions and optimization techniques were applied to achieve the best possible performance. The inter-reference memory organization allowed for efficient use of global memory, while the block-based approach was devised to exploit on-chip shared memory. The block-based implementation gave rise to applying loop unrolling and data compression, which further improved performance. Apart from the thoroughly optimized OpenCL implementation, we also developed a SSE4.1 vectorized version of the kernel to investigate how state-of-the-art SIMD platforms perform for PaPaRa. Via the competing programmer approach, we hope to provide an *as fair as possible* performance comparison between two fundamentally different hardware architectures. On an Intel i7 2600 CPU (using all 4 physical cores), we obtained a x86 SIMD peak performance of 18 GCUPS and an average performance of 12.3 GCUPS. A mid-range gaming GPU like the GTX 560 delivered peak and average performance of 22.1 and 18.4 GCUPS.

We developed and optimized the OpenCL kernel mainly for the NVIDIA Fermi GPU architecture. Nonetheless, we also executed some exploratory tests on an AMD/ATI GPU (a RADEON HD 6970 with a theoretical peak performance of 2703 GFLOPS). As expected, the OpenCL kernel could be executed on the ATI system without any substantial modifications, but we observed poor performance. We measured performance of 13.2 GCUPS (the NVIDIA GTX 560 GPU with 1075 GFLOPS peak performance delivered 18.9 GCUPS on this dataset) on a subset of the real-world dataset used to evaluate the performance of the hybrid CPU-GPU system. One would expect a 3 to 4 times better GCUPS performance for a fully ATI-tuned kernel. Note however that, in contrast to the NVIDIA Fermi architecture, the AMD/ATI architecture heavily relies on Instruction Level Parallelism (ILP). Thus, it may become necessary to re-write the alignment kernel such that the OpenCL compiler can group similar instructions more efficiently in an SIMD-like manner to attain peak performance. Thus, while OpenCL code is portable, achieving satisfying performance still requires an architecture-aware tuning.

Finally, we combined and coupled the SIMD and SIMT implementations to create a hybrid CPU-GPU system that can now exploit all available computational resources of a representative modern desktop system (GTX 560 GPU and i7 2600 CPU). The total system peak performance amounts to 33.8 GCUPS. By efficiently exploiting all computational resources, we are able to fundamentally improve the applicability of the highly accurate PaPaRa algorithm to large real-world datasets. For dataset 16S.B.ALL, the major concern regarding PaPaRa expressed in [19] is the relatively long program runtimes of the original (unvectorized and unoptimized) proof-of-concept implementation. In terms of alignment quality, PaPaRa outperforms all alternative approaches that have been assessed on large real-world datasets according to this independent study. To test the current limits of CPU performance using PaPaRa, we conducted additional tests on an overclocked system (Intel i5 2500k CPU running at 4.5 GHz). This type of CPU has an unlocked clock-frequency generator and can therefore be overclocked without additional hardware modifications, except for providing appropriate cooling. On the overclocked CPU, we measured a peak performance of 21.9 GUPS on 4 cores (using 1250 QS and 320 RS of length 1000). This corresponds to an improvement of 20% over the i7 2600 CPU. We did not experience any stability issues for the specific overclocking configuration during these tests. Based on the performance data published at http://www.spec.org/cpu2006/results/cpu2006.html, we conclude that this CPU, when pushed to its technical limits, currently offers the highest per-core performance of any available CPU on the market. Finally, our results corroborate the observations made in [9] that the computational capabilities of modern CPUs and GPUs are in the same order of magnitude provided that highly optimized alignment kernels are developed on both platforms.

## Conclusions

In this paper we described the adaptation and acceleration of a novel phylogeny-aware short-read alignment kernel named PaPaRa to modern x86 and SIMT architectures. For the SIMT architecture we used OpenCL while for the SIMD platform we deployed multi-threading and SSE4.1 vector intrinsics. We observed that state-of-the-art CPUs and GPUs deliver comparable performance for sequence alignment algorithms if properly optimized. We also demonstrated that overall system performance can be substantially improved when all computational resources are used (CPU and GPU).

The SIMD and SIMT implementations were developed using the “competing programmer approach”. Thus, the programming time and tuning effort spent on both implementations was comparable. The performance of the resulting codes is analogous to the performance obtained for previous SIMD and SIMT accelerations of the related, yet not identical SWA. We conclude that, for representative dynamic programming kernels, deploying SIMD vector intrinsics is as challenging as porting the algorithm to an SIMT platform. In both cases, a thorough understanding of the underlying hardware architecture is required (also with respect to performance results on the AMD/ATI platform). An understanding of CPU architectures can help to reduce cache misses and/or pipeline stalls. Understanding how threads are launched on a GPU can help to reduce/eliminate memory access conflicts among parallel threads and therefore increase the number of executed instructions per cycle.

Regarding future work, we intend to also explore CUDA as an alternative to OpenCL as well as to devise an analogous performance comparison. Furthermore, we plan to investigate how a OpenCL code, as optimized for a GPU, performs on multi-core CPUs. We also intend to analyze the programming effort that is required to transform a GPU-optimized OpenCL implementation into a CPU-optimized one. Finally, we plan to implement a block-based CPU version of the kernel for further reducing cache misses and for improving CPU performance on very large reference sequences.

## Abbreviations

SIMD: Single Instruction Multiple Data; MIMD: Multiple Instruction Multiple Data; SIMT: Single Instruction Multiple Threads; PaPaRa: PArsimony-based Phylogeny-Aware short Read Alignment; GCUPS: Giga Cell Updates Per Second; MSA: Multiple Sequence Alignment; QS: Query Sequence, RS, Reference Sequence; RT: Reference Tree; SWA: Smith-Waterman Algorithm; FPGA: Field-Programmable Gate Array; OpenCL: Open Computing Language; CUDA: Compute Unified Device Architecture.

## Competing interests

The authors declare that they have no competing interests.

## Authors’ contributions

NA, AS and SB designed the study. NA implemented the OpenCL code for the SIMT platform. S.B. implemented the vectorized C code for the SIMD platform. NA, SB and AS wrote and edited the manuscript. All authors read and approved the final manuscript.
